# *Hermetia illucens,* an innovative and sustainable source of chitosan-based coating for postharvest preservation of strawberries

**DOI:** 10.1016/j.isci.2023.108576

**Published:** 2023-11-24

**Authors:** Micaela Triunfo, Anna Guarnieri, Dolores Ianniciello, Leonardo Coviello, Antonella Vitti, Maria Nuzzaci, Rosanna Salvia, Carmen Scieuzo, Patrizia Falabella

**Affiliations:** 1 Department of Sciences, University of Basilicata, Via dell’Ateneo Lucano 10, 85100 Potenza, Italy; 2 School of Agricultural, Forestry, Food and Environmental Sciences, University of Basilicata, Via dell’Ateneo Lucano 10, 85100 Potenza, Italy; 3 Spinoff XFlies s.r.l, University of Basilicata, Via dell'Ateneo Lucano 10, 85100 Potenza, Italy

**Keywords:** Microbiology, Applied microbiology, Biotechnology, Food technology

## Abstract

The ability of chitosan produced from pupal exuviae of *Hermetia illucens* to retard the decay of the local strawberry (*Fragaria x ananassa)* cultivar *Melissa* was investigated for the first time in this paper. The results demonstrated the effectiveness of insect chitosan compared to the commercial polymer in preserving and enhancing, at the same time, some physicochemical parameters (weight loss, pH and soluble solids content) and nutraceutical properties (total polyphenol content, total flavonoid content and total antioxidant activity) of strawberries stored at RT, 4°C and at mixed storage conditions (4°C + RT). Moreover, chitosan from *H. illucens* was also effective in reducing fungal decay and improving fruit shelf life. The obtained results confirm that insect chitosan, particularly deriving from *H. illucens* pupal exuviae, can be a viable alternative to crustacean one in safeguarding postharvest fruits.

## Highlights

• The rapid perishability of fresh foods is one of the main causes of food waste
• A sustainable solution is edible coatings obtained from ecofriendly biomaterials
• Chitosan from *Hermetia illucens* slows down the decay of strawberries

## Graphical abstract

**Figure:**
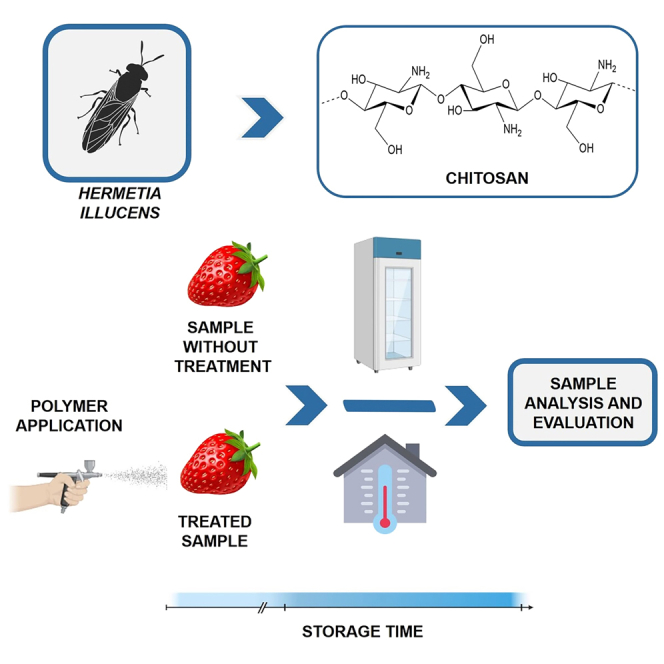

## Introduction

One of the most important needs of the worldwide food industry involves keeping food for consumption fresh or minimally processed.^1^ Strawberries are particularly widespread worldwide as a seasonal fruit, as well as the most widely consumed berries. They are rich in vitamin A, vitamin B and vitamin C, but also in minerals such as potassium, calcium, magnesium, sulfur and iron, as well as amino acids.^2^ They are highly appreciated because they are rich in important organoleptic properties related to the content of antioxidant agents such as flavonoids, anthocyanins, and phenolic acids.^3^ Consistency, taste (related to sugar and organic acid content), flavor and color are important aspects that denote the quality of these fruits. Several factors, including pre- and postharvest environmental conditions and genotype, influence their phytochemical composition.^4^ However, strawberries are a non-climacteric fruit, with a postharvest life not being particularly long-lasting, placing them in a condition of very rapid decline.^3^^,^^5^ The perishability of this fruit is mainly related to a remarkably rapid and proactive metabolism,^6^ as well as a heightened susceptibility to several fungal attacks, causing microbial spoilage such as *Botrytis cinerea* and *Rhizopus* spp.^5^ Furthermore, their rapid deterioration is also related to susceptibility to water loss, causing softening, and also to mechanical damage that is dependent on their particularly smoothness firmness, mainly due to the absence of an external protective shield.^5^ The attacks by pathogens commonly occur in various steps of transportation, preservation and storage of the fruits.^7^ In order to cope with this issue, numerous preservation practices have been studied such as osmotic and hypobaric treatments, preservation under refrigeration, then by cold, via irradiation, and in a modified and controlled atmosphere.^8^^,^^9^^,^^10^ This last technique employs oxygen at a low concentration and carbon dioxide at a high concentration, both useful for countering strawberry decay and inhibiting the growth of pathogens.^11^^,^^12^^,^^13^ However, globally, alternative strategies are being pursued in order to limit the spoilage of fresh postharvest foods with the aim of both reducing decay and protecting human health. Edible coatings are of great interest, as they can also carry biologically active substances that enhance fruit storage.^14^ The mechanism of action of these coatings makes it possible to maintain the quality of processed fruits, providing a semi-permeable barrier to volatile compounds, moisture vapor and gases. In addition, they also play a role in preserving the integrity of the product, both structurally and mechanically.^15^ Edible coating materials can vary in their nature, and they can be lipid-, protein-, and polysaccharide-based.^16^^,^^17^ One of the edible coating polysaccharides of major interest is chitosan, due mainly to its antifungal and antimicrobial properties, that are functional in enhancing the shelf life of fresh fruit.^18^^,^^19^^,^^20^^,^^21^^,^^22^^,^^23^ Chitosan is able to stimulate plant defenses and prevent disease development.^24^ Indeed, it was approved as a basic substance by the European Union for plant protection purposes, according to Regulation (EU) No 563/2014, for both organic agriculture and integrated pest management. Many studies have reported the effectiveness of 1% chitosan in reduction of disease incidence against phytopathogenic fungi, such as gray mold and *Rhizopus* rot, in postharvest decay of fresh fruit.^25^ At the commercial scale, chitosan is produced by the deacetylation of chitin extracted from crustaceans.^26^ The growing market demand for this biopolymer led researchers searching for alternative sources to crustaceans, susceptible to seasonality and to geographical limitations, that could ensure a steady supply. The key solution was found in insects, whose exoskeleton is rich in chitin.^27^^,^^28^ Among insects, *Hermetia illucens*, the black soldier fly, has particular relevance, and it is bred widely in many European states.^29^^,^^30^ *H*. *illucens* larvae feed on waste from the food supply chain, converting it into larval biomass, rich in molecules of high-biological value, including chitin.^31^^,^^32^^,^^33^^,^^34^ Chitin can be extracted from various biomass from *H*. *illucens*, such as larvae, pupal exuviae and dead adults. Pupal exuviae, a waste product of insect breeding, represent the biomass of choice for the extraction of polymer, containing about 25% chitin, which can be processed to produce chitosan.^27^^,^^28^ Properly solubilized in acid medium, chitosan can be sprayed on fruits, in order to obtain a functional coating for their preservation.^7^^,^^18^^,^^35^^,^^36^

The aim of research is to use and to investigate for the first time the chitosan produced from insects, particularly from the pupal exuviae of *H*. *illucens*, as a preservative coating for a new Lucanian strawberry (*Fragaria x ananassa), cv.* “*Melissa”.* The perishability of this new strawberry variety has been studied for the first time. There are several studies discussing the effect of commercial chitosan derived from crustaceans and used for fresh fruit preservation. In contrast, there are few studies evaluating the effect of insect chitosan. By comparison with commercial chitosan, insect polymer, in particular from *H. illucens,* has proved to be a viable and effective alternative in preserving the decay of the strawberry varieties tested.

## Results and discussion

### Chemical characterization of chitosan

Deacetylation degree (DD) and viscosity-average molecular weight (M_v_) of not decolorized (No Dec) and decolorized (Dec) chitosan from pupal exuviae obtained from *H. illucens* were determined. Insect chitosan showed the same DD values as crustacean-derived chitosan, falling in a range of 85–90%, as reported in other of our papers.^35^^,^^36^ The M_v_ of all chitosan samples produced from *H. illucens* was much lower (75 and 150 kDa, for Dec and No Dec chitosan from pupal exuviae, respectively) than that of commercial chitosan (about 370 kDa).^36^ The Mw values calculated for chitosan from *H. illucens* are within the range reported for insect chitosan (30–300 kDa).^26^ It is generally reported that chitosan with low Mw (<150 kDa) has higher antibacterial activity than chitosan with high Mw, due to the ease of crossing the bacterial cell wall.^21^

### Antioxidant activity of chitosan

**Figure 1:**
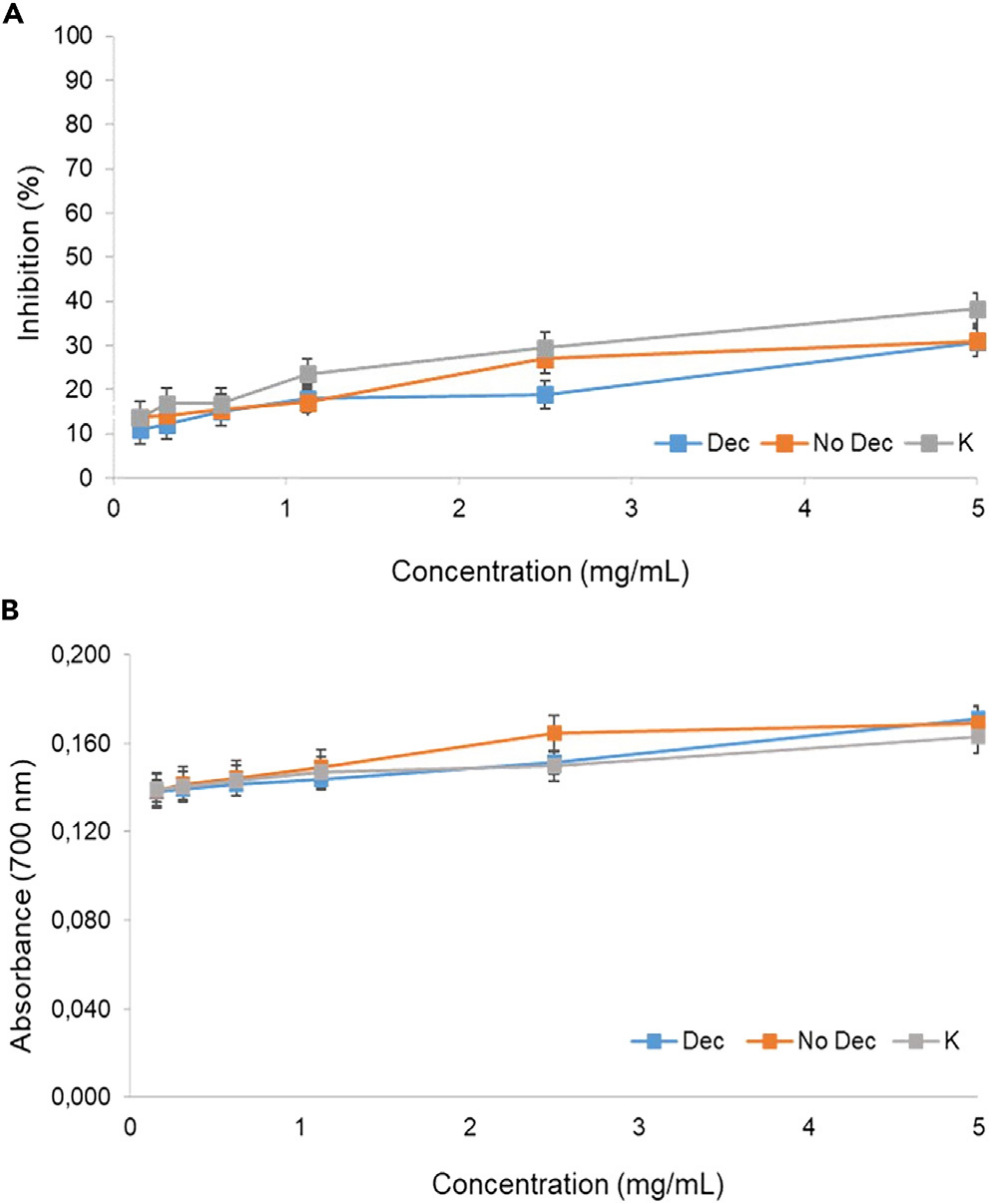
Chitosan free radical scavenging activity and ferric reducing power Free radical scavenging activity (A) and ferric reducing power (B) of not decolorized (No Dec) and decolorized (Dec) chitosan samples obtained from *H. illucens* pupal exuviae and commercial one derived from crustaceans (K). Data are expressed as mean ± standard deviation (n = 3).

The antioxidant abilities of No Dec and Dec chitosan from pupal exuviae obtained from *H. illucens* were assessed. Results were reported in Table 1 and Figures 1A and 1B. As expected, for all tested chitosan samples, the radical scavenging effect increased with increasing concentrations tested (Figure 1A). Chitosan from *H. illucens* showed good free radical scavenging activity, with IC_50_ values of 10.32 and 9.66 mg/mL for No Dec and Dec chitosan, respectively, similar to those estimated for commercial chitosan (K), but higher than ascorbic acid (0.33 mg/mL), the reference standard with the highest antioxidant activity (Table 1). At 5 mg/mL, all chitosan exhibited radical scavenging activity between 30 and 32%, similar on average to the commercial sample (38%) (Figure 1A). The reducing power of the tested chitosan samples also showed a slightly increasing and concentration-dependent trend, with the chitosan from *H. illucens* better than crustacean-derived chitosan. As expected, the standard antioxidant used was the most effective in reducing the Fe^3+^/Ferricyanide complex (Figure 1B). At 5 mg/mL, the reducing power of the chitosan from *H. illucens* was about 0.170 abs, a comparable absorbance value and slightly higher than that measured for the commercial sample (0.160 abs). No Dec and Dec chitosan from pupal exuviae showed a good reducing power, with EC_50_ values of 63 and 53 mg/mL, respectively, significantly lower and better than the 77 mg/mL estimated for the commercial sample (Table 1). Due to the lack of studies on the antioxidant activity of chitosan produced from *H. illucens*, it was not possible to compare our results with others in the literature. Particularly, chitosan from *H. illucens* showed IC_50_ values similar to those reported for chitosan from shiitake fungi and crab shells (9.13–16.30 mg/mL),^37^^,^^38^ for chitosan from grasshoppers (*Calliptamus barbarus* and *Oedaleus decorus*) that were around 11 mg/mL^39^ and for that from larvae of *Leptinotarsa decemlineata* (10.4 mg/mL).^39^ In contrast, our IC_50_ values were higher than those reported for adults of *L. decemlineata* (4.15 mg/mL) and for larvae of *Musca domestica* (2–4 mg/mL).^39^^,^^40^ Better activity was found compared to chitosan from eggs of the crustacean *Daphnia longispina* (23–56.4 mg/mL) and chitosan from *Zophobas morio* larvae (65.9–140.7 mg/mL).^41^^,^^42^ For the reducing activity, all chitosan samples showed higher EC_50_ values, and thus low efficacy, than those reported by chitosan from crustaceans and insects, ranging from 4.5 mg/mL, for chitosan from *L. decemlineata*, to about 30 mg/mL, for chitosan from grasshoppers.^39^^,^^43^

**Table 1:** Scavenging ability and ferric reducing activity of chitosan

SAMPLE	DPPH	FRAP
	IC_50_ (mg/mL)	EC_50_ (mg/mL)
No Dec	10.32 ± 1.8^b^	63.02 ± 4.5^b^
Dec	9.66 ± 2.2^b^	52.78 ± 4.7^b^
K	7.38 ± 1.2^b^	76.83 ± 3.9^c^
Ascorbic acid	0.33 ± 1.2^a^	0.04 ± 3.4^a^

Scavenging ability and ferric reducing activity of not decolorized (No Dec) and decolorized (Dec) chitosan samples obtained from *H. illucens* pupal exuviae, of the commercial one derived from crustaceans (K) and of the standard (Ascorbic acid). Data are expressed as mean ± standard deviation (n = 3). Different letters in a column indicate significant differences in the IC_50_ and EC_50_ among the chitosan samples (p < 0.001) (data analyzed with one-way ANOVA and Tuckey *post-hoc* test).

Properties such as Mw and DD were reported to influence the antioxidant activity of chitosan. Specifically, high DD values showed to promote a more efficient scavenging action; for Mw, on the other hand, the lower its value, the higher the antioxidant activity, probably related to the smaller polymer chain size, which formed fewer intramolecular hydrogen bonds, thus leaving the reactive groups more accessible and functional for radical scavenging activity.^44^ This is in line with the properties of chitosan from *H*. *illucens*, already demonstrated by our research group.^35^^,^^36^ Indeed, our chitosan with a lower Mw, was found to be better than commercial chitosan, characterized by a higher Mw.^27^^,^^35^

### Effect of chitosan coating on weight loss of strawberry fruit

**Figure 2:**
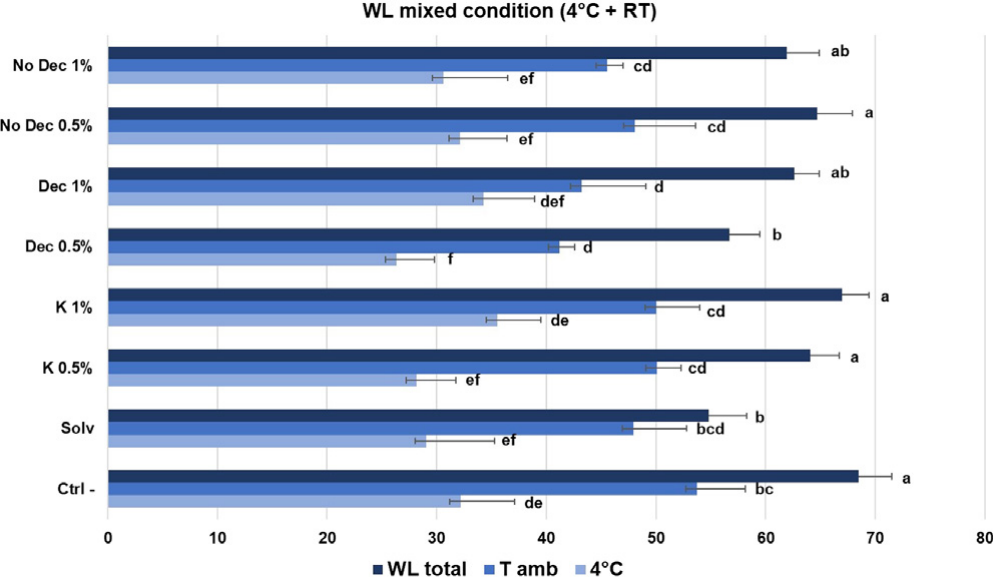
Strawberries weight loss evaluation Evaluation of weight loss (WL) during the two storage temperatures (cold storage and RT) individually considered of the mixed condition (4°C + RT). Conditions: total weight loss (dark blue bands), weight loss for the first 7 days at 4°C (light blue bands) and for the last 3 days at RT (blue bands). Treatments: negative control (Ctrl -), solvent (solv), coating with pupal exuviae not decolorized (No Dec), decolorized (Dec) and commercial (K) chitosan, both at 0.5% and 1%. Data are expressed as mean ± standard deviation (n = 5). Different letters indicate significant differences among the treatments for each storage temperature of the mixed condition (p < 0.05) (Data analyzed with one-way ANOVA and Tuckey *post-hoc* test).

Fruit weight loss is an important index reflecting the respiration rate and the moisture evaporation between the fruit tissue and the surrounding air. Chitosan-based edible coatings act as a barrier by forming a semi-permeable layer on the fruit surface that reduces transpiration losses.^45^ During postharvest storage, significant decreases in fruit firmness occur due to biochemical changes in cell turgor and in the structure and composition of cell wall polysaccharides.^5^ Strawberry fruits become softer as the middle lamella degrades and pectin decreases during postharvest storage. Therefore, the decline in firmness is often considered a sign of decreasing quality or microbial attack.^46^ After 4 days of storage at RT, strawberries coated with chitosan from *H. illucens*, both No Dec and Dec, revealed significantly lower weight losses compared to the solvent-coated fruit and negative control. Specifically, Dec samples (both 0.5% and 1%) were particularly effective coating solutions, especially compared to commercial chitosan (Table 2). Benhabiles et al. reported comparable results for treatments with commercial crustacean-derived chitosan; therefore, our results with insect chitosan are very promising.^45^ After 14 days of storage at 4°C, on the other hand, a significant reduction in the weight loss was observed only in strawberries treated with Dec chitosan samples from *H. illucens*; particularly the lowest loss was found in fruits coated with Dec 0.5% chitosan. At this storage condition, the coating with the No Dec chitosan samples (both 0.5% and 1%), however, showed a similar effect compared to the treatment with commercial chitosan at both tested concentrations (Table 2). These results are in agreement with literature reports for commercial chitosan.^18^^,^^19^ Particularly, at 4°C, as also observed by Petriccione et al., the effect of commercial chitosan appears to be strongly correlated to the concentration.^18^ Indeed, commercial chitosan was more effective at the highest tested concentration (2%). In contrast, for insect chitosan, the best activity was obtained with Dec chitosan treatments, compared to No Dec ones, already at 0.5%; therefore, the effect of chitosan from *H. illucens* might seem to be related more to the chemical features of the polymer resulting from the purification process, than to the tested concentration.^27^^,^^35^ At the end of mixed storage condition, which included a period of 7 days at 4°C, followed by a period of 3 days at RT (4°C + RT), Dec chitosan samples, particularly Dec 0.5%, as well as No Dec 1% chitosan, were effective in significant reduction of weight loss compared to both the negative control and the commercial chitosan coating (Table 2). Specifically, for the mixed storage condition (4°C + RT), in addition to the total, the partial weight loss of strawberries during the days stored at 4°C and the days at RT was evaluated separately, as shown in Figure 2. As expected, after one week at controlled temperature, the weight loss was moderate amounting to about 31%. The lowest rates (below 30%) were measured for strawberries coated with Dec 0.5% chitosan from *H. illucens* (26%), statistically significant compared to K1% (35.5%, the highest WL value). These values were increased by moving the strawberries to RT, with an average weight loss of 48%. In this case, significant differences compared to the negative control were only measured for Dec chitosan, at both concentrations tested (41% and 43% for Dec 0.5 and 1%, respectively). These values, although not statistically significant, were followed by those of No Dec chitosan, with lower values (48% and 46% for No Dec 0.5% and 1%, respectively) compared to the treatments with K sample, which were around 50%. The negative control showed the highest moisture losses (53%). The results obtained in these experimental conditions, which showed a good activity even for No Dec chitosan, were in accordance with those of Petriccione et al*.*^18^ Indeed, at the highest tested concentration (1%), Dec chitosan was more efficient than No Dec chitosan. This higher activity of Dec chitosan compared to No Dec one from *H. illucens* could be attributed to the bleaching step of the chitin resulting in a chitosan with a lower Mw.^35^ The positive effect of chitosan coating was demonstrated on many other fruits, such as blueberry,^47^ grape,^48^ mango,^49^ papaya.^50^ The solvent-only coating, however, gave a similar weight loss to the negative control at RT. This could be due to a solvent composition that may not reduce fruit transpiration and thus weight loss. However, it is encouraging to note that the solvent addition of chitosan, particularly from insects, enhanced the effect, resulting in less weight loss than the negative control.

**Table 2:** Results of evaluation of weight loss, soluble solids content and pH of treated and untreated strawberries

Treatments	WL (%)	SSC (°Brix)	pH
Before treatment		9.2 ± 0.3	3.42 ± 0.05

**RT**			

Ctrl -	53.6 ± 3.1^a^	13.6 ± 0.9^ab^	3.22 ± 0.01^a^
Solvent	54.2 ± 3.2^a^	14.8 ± 1.0^a^	3.25 ± 0.04^a^
K 0.5%	49.1 ± 2.8^ab^	15.9 ± 0.4^a^	3.24 ± 0.04^a^
K 1%	51.5 ± 2.0^a^	16.3 ± 1.9^a^	3.25 ± 0.01^a^
Dec 0.5%	42.7 ± 2.5^b^	14.3 ± 0.2^ab^	3.11 ± 0.06^b^
Dec 1%	43.3 ± 2.9^b^	11.4 ± 1.9^b^	3.20 ± 0.01^ab^
No Dec 0.5%	48.1 ± 2.9^ab^	13.6 ± 0.6^ab^	3.21 ± 0.04^a^
No Dec 1%	49.7 ± 1.8^ab^	14.2 ± 0.4^ab^	3.22 ± 0.01^a^

**4°C**			

Ctrl -	52.4 ± 2.8^ab^	14.7 ± 0.6^a^	3.38 ± 0.05^a^
Solvent	55 ± 3.2^a^	14.7 ± 0.5^a^	3.34 ± 0.07^a^
K 0.5%	55.4 ± 3.0^a^	15.4 ± 0.4^a^	3.37 ± 0.07^a^
K 1%	50.8 ± 1.4^ab^	15.5 ± 0.3^a^	3.39 ± 0.07^a^
Dec 0.5%	47.4 ± 1.6^b^	13.2 ± 0.4^b^	3.36 ± 0.10^a^
Dec 1%	49.2 ± 1.5^b^	12.7 ± 0.1^b^	3.34 ± 0.15^a^
No Dec 0.5%	55.4 ± 2.7^a^	13.7 ± 0.5^b^	3.42 ± 0.10^a^
No Dec 1%	55 ± 2.6^a^	13.3 ± 0.4^b^	3.33 ± 0.14^a^

**4°C + RT**			

Ctrl -	68.5 ± 3.0^a^	19.±1.2^a^	3.41 ± 0.04^a^
Solvent	54.8 ± 3.5^b^	14.4 ± 1.2^bc^	3.38 ± 0.04^a^
K 0.5%	64.1 ± 2.6^a^	14.7 ± 0.3^bc^	3.37 ± 0.04^a^
K 1%	67 ± 2.4^a^	16.9 ± 0.3^a^	3.39 ± 0.06^a^
Dec 0.5%	56.7 ± 2.8^b^	16.4 ± 0.6^b^	3.37 ± 0.02^a^
Dec 1%	62.6 ± 2.3^ab^	14.1 ± 0.3^c^	3.38 ± 0.06^a^
No Dec 0.5%	64.7 ± 3.2^a^	16.3 ± 1.1^b^	3.40 ± 0.01^a^
No Dec 1%	61.9 ± 3.0^ab^	14.3 ± 0.3^bc^	3.42 ± 0.06^a^

Results of evaluation of weight loss (WL), soluble solids content (SCC) content and pH on strawberries stored at RT, 4°C and mixed condition (4°C + RT). Treatments: negative control (Ctrl -), solvent, coating with pupal exuviae not decolorized (No Dec), decolorized (Dec) and commercial (K) chitosan, both at 0.5% and 1%. Data are expressed as mean ± standard deviation (n = 5). Means followed by different letters in the column are significantly different (p < 0.05) by one-way ANOVA and Tuckey *post-hoc* test.

### Effect of chitosan coating on soluble solids content of strawberry fruit

One index of fruit ripeness is the soluble solids content (SSC), that represents a sugar content estimation. The conversion of fruit starch to sugars such as fructose, glucose, and saccharose results in the SSC increasing over time during maturation due to a hydrolytic reaction.^51^ The increase in total soluble solids content could be related to various pathways, such as decreasing the rate of respiration, loss of water, and destruction of the cell wall.^10^ In our experiment, SSC of chitosan-treated and untreated strawberries increased during storage for all analyzed conditions (Table 2), as reported by Taha et al. in which crustacean chitosan was used to improve strawberry shelf-life.^52^ Particularly, in strawberries stored at 4°C and 4°C + RT, chitosan from *H*. *illucens* was effective in containing the increase in SSC; Dec 1% chitosan proved to be the most powerful in limiting the SSC increase during storage, and it was also statistically significant compared to the negative control (Table 2). Our results are better than those obtained by Taha et al.*,*^52^ where chitosan was statistically significant only compared to the negative control, in contrast to our chitosan samples which at RT was found to be effective treatment even compared to the solvent and to the commercial chitosan.

At 4°C after 2 weeks of storage, all chitosan-based coatings from *H*. *illucens*, both No Dec and Dec samples, gave a significant reduction in the SSC increase compared to the negative control, the solvent-coated fruit and commercial chitosan (Table 2), in agreement with Taha et al*.*^52^ This result is also compatible with the work of Petriccione et al., that showed that uncoated strawberry varieties "*Jonica*" and "*Sabrina*" exhibited a considerably higher increase of SSC than the chitosan-treated fruits.^18^ Other works also reported positive effects of chitosan treatment on other fruits, such as pomegranates, apples and papaya.^50^^,^^53^^,^^54^ The mix of temperature conditions (4°C + RT) is the best storage conditions to highlight the potential of the polymer in controlling this process; indeed, chitosan from *H. illucens* was statistically significant compared to the negative control and K1% chitosan; specifically, at the highest tested concentration (1%), insect chitosan samples, both No Dec and Dec, proved to be the most effective in maintaining the increase in SSC (Table 2). The results obtained from our experiments, therefore demonstrated the effectiveness of chitosan coatings from *H. illucens* in reducing the increase in SSC, comparing them to both negative control and solvent. This is in accordance with literature reports.^18^^,^^52^^,^^55^ In some instances, our treatments were also better than commercial crustacean-derived chitosan. However, it is important to specify that a direct comparison with works on chitosan-coated strawberries from *H. illucens* is not possible, as this is the first work where the insect polymer was employed on this fruit species. The biopolymer, indeed, could directly act on the internal atmosphere of the fruit. The mechanism of action may involve a reduction in O_2_ levels and an increase in CO_2_ levels and, as a result, the rate of respiration and metabolic activity is reduced.^49^

### Effect of chitosan coating on pH variation in strawberry fruit

Inside the fruits, the pH variation is mainly related to the content of organic acids.^19^ These are substrates used by enzymes involved in fruit respiration, which accumulate during the maturation period.^52^ The conversion of acid and starch to sugars results in the fruit acidity decreasing during postharvest storage. This occurs through acid metabolism.^47^ The acid content in fruit is directly related to glycolytic metabolism and the tricarboxylic acid cycle, accelerated via the increased rate of respiration.^56^ However, it should be noted that the acidity of the fruit is also related to factors such as the variety and its degree of maturation. Alongside this, there are also other parameters such as the geographical areas of crop distribution, the growing climate and the transport conditions.^57^ In our experiment, the pH of the strawberries remained rather stable during storage under all conditions tested. Only in strawberries kept at RT, all Dec chitosan samples gave a significantly smaller increase in pH than the negative control and the solvent, showing a better action even compared to commercial chitosan. Concretely, the Dec 0.5% chitosan was found to be the most effective solution (Table 2). At 4°C, none of the applied treatment significantly reduced the pH increase compared to the negative control. Although not significant, Dec chitosan (both 0.5 and 1%) and No Dec 1% chitosan had a better effect than the negative control (Table 2). At the mixed storage condition (4°C + RT), the Dec chitosan treatments were able to not increase pH values, although not in a significant manner, than negative control (Table 2). The maintenance of pH values on the same fruits stored at RT, exploiting crustacean chitosan, was detected by Velickova et al.^58^ and Ali et al*.*^3^ Jiang et al. highlighted differences between untreated and treated strawberries stored at 4°C, for which the pH increased very slightly compared to the variation in the untreated control.^19^ Similar results were also obtained by Quintana et al. demonstrating the effectiveness of crustacean chitosan added with active compounds in the storage of strawberries, compared to unprocessed fruit.^59^ Benhabiles et al., on the other hand, observed no significant differences for coated strawberries at RT;^45^ probably the increase in pH was also related to the ripening stage of the fruit. Similar results were also obtained for other fruits coated with commercial chitosan.^60^^,^^61^ The control of the pH increase mediated by the crustacean-derived polymer was effective for several fruits, such as blueberries, mangoes and plums.^47^^,^^51^^,^^62^

### Effect of chitosan coating on nutraceutical compounds and antioxidant activity of strawberry fruit

Many papers were published on the abilities of crustacean-derived chitosan coating to preserve phenolic and flavonoid compounds and antioxidant activity in fruits. To the best of our knowledge, the effect of insect-derived chitosan, however, was not yet tested on fruits and vegetables; there is only a preliminary investigation on preservation of fresh cherry tomatoes and *Prunus* species fruits, also carried out by our research group.^35^^,^^36^ Changes in nutraceutical properties were evaluated by quantifying the total phenols (TP),^35^ total flavonoids (TF),^63^ total anthocyanins (TA)^64^^,^^65^ and total antioxidant activity (TAA)^66^ of new local cultivar of strawberry *cv. “Melissa”*, as reported in Table 3.

**Table 3:** Results of evaluation of total phenol content, total flavonoid content, total antioxidant activity and total anthocyanins of treated and untreated strawberries

Treatments	TP (mg GAE g^−1^ FW)	TF (mg QUE g^−1^ FW)	TAA (mg TE g^−1^ FW)	TA (mg P3g g^−1^ FW)
Before treatment	1.84 ± 0.03	0.165 ± 0.004	4.45 ± 0.59	0.201 ± 0.04

**RT**				

Ctrl -	3.44 ± 0.05^c^	0.268 ± 0.02^a^	10.75 ± 0.01^bc^	0.493 ± 0.11^a^
Solvent	3.27 ± 0.05^d^	0.237 ± 0.04^a^	9.50 ± 0.11^e^	0.512 ± 0.14^a^
K 0.5%	3.81 ± 0.04^ab^	0.247 ± 0.04^a^	10.24 ± 0.17^cd^	0.512 ± 0.07^a^
K 1%	3.91 ± 0.04^a^	0.236 ± 0.04^a^	11.44 ± 0.33^a^	0.524 ± 0.11^a^
Dec 0.5%	3.39 ± 0.07^cd^	0.228 ± 0.01^a^	9.84 ± 0.13^de^	0.534 ± 0.07^a^
Dec 1%	3.31 ± 0.05^cd^	0.276 ± 0.04^a^	9.34 ± 0.29^e^	0.528 ± 0.09^a^
No Dec 0.5%	3.76 ± 0.02^b^	0.261 ± 0.02^a^	9.88 ± 0.25^de^	0.512 ± 0.07^a^
No Dec 1%	3.86 ± 0.03^ab^	0.257 ± 0.03^a^	11.15 ± 0.25^ab^	0.545 ± 0.07^a^

**4°C**				

Ctrl -	3.42 ± 0.04^b^	0.233 ± 0.04^a^	9.65 ± 0.10^b^	0.513 ± 0.02^a^
Solvent	2.64 ± 0.04^e^	0.218 ± 0.04^a^	8.30 ± 0.27^c^	0.586 ± 0.02^a^
K 0.5%	2.79 ± 0.06^d^	0.225 ± 0.01^a^	8.83 ± 0.33^c^	0.563 ± 0.04^a^
K 1%	2.89 ± 0.05^d^	0.230 ± 0.01^a^	8.84 ± 0.33^c^	0.541 ± 0.01^a^
Dec 0.5%	2.62 ± 0.05^e^	0.224 ± 0.01^a^	9.27 ± 0.28^bc^	0.515 ± 0.04^a^
Dec 1%	3.51 ± 0.05^ab^	0.263 ± 0.01^a^	9.66 ± 0.22^b^	0.578 ± 0.10^a^
No Dec 0.5%	3.22 ± 0.04^c^	0.238 ± 0.01^a^	9.68 ± 0.23^b^	0.560 ± 0.07^a^
No Dec 1%	3.58 ± 0.04^a^	0.258 ± 0.01^a^	10.83 ± 0.36^a^	0.556 ± 0.10^a^

**4°C + RT**				

Ctrl -	3.38 ± 0.05^ef^	0.260 ± 0.01^bc^	9.15 ± 0.20^e^	0.507 ± 0.12^b^
Solvent	3.47 ± 0.05^de^	0.242 ± 0.04^c^	9.10 ± 0.14^e^	0.541 ± 0.01^b^
K 0.5%	3.24 ± 0.05^fg^	0.248 ± 0.01^c^	8.63 ± 0.22^e^	0.506 ± 0.10^b^
K 1%	3.07 ± 0.07^g^	0.251 ± 0.01^bc^	7.00 ± 0.02^f^	0.503 ± 0.10^b^
Dec 0.5%	3.76 ± 0.07^bc^	0.342 ± 0.03^a^	10.55 ± 0.35^c^	0.643 ± 0.04^ab^
Dec 1%	3.88 ± 0.1^b^	0.328 ± 0.03^ab^	11.41 ± 0.10^b^	0.709 ± 0.10^ab^
No Dec 0.5%	4.32 ± 0.1^a^	0.352 ± 0.04^a^	12.45 ± 0.31^a^	0.718 ± 0.08^ab^
No Dec 1%	3.60 ± 0.04^cd^	0.356 ± 0.01^a^	9.78 ± 0.11^d^	0.799 ± 0.08^a^

Results of evaluation of total phenols (TP), total flavonoids (TF), total antioxidant activity (TAA) and total anthocyanins (TA) on strawberries stored at RT, 4°C and mixed condition (4°C + RT). Treatments: negative control (Ctrl -), solvent, coating with pupal exuviae not decolorized (No Dec), decolorized (Dec) and commercial (K) chitosan, both at 0.5% and 1%. Data are expressed as mean ± standard deviation (n = 5). Means followed by different letters in the column are significantly different (p < 0.05) by one-way ANOVA and Tuckey *post-hoc* test.

#### Total phenol content

The Lucanian strawberry cultivar "*Melissa*" studied for the first time in this work, exhibited at harvest a total phenol content (TPC) of 1.84 mg GAE g^−1^, a value comparable to that measured by Petriccione et al. for "*Jonica*" (1.94 mg GAE g^−1^), the geographically closest cultivar.^18^ The accumulation of phenolic compounds, as well as flavonoid ones, in addition to fruit variety and ripening stage, can be influenced by other crop-dependent factors, such as genetic background, climatic conditions, and agronomic practices.^4^^,^^67^ The application of the different coating treatments induced an increase in the TPC of the strawberries for all storage conditions tested, with a significant TPC that ranged from 3.2 to over 4 mg GAE g^−1^, depending on the concentration and storage conditions. Strawberries stored at RT, after 4 days showed less pronounced differences between treatments, with the solvent solution exhibiting the lowest TPC, compared to negative control. Treatments with both K and No Dec chitosan, on the other hand, provided phenol recovery by showing the highest contents, with slightly better performance for the 1% coating solutions (Table 3). Dec chitosan had intermediate TPC and were similar to the negative control (Table 3). When the strawberries were stored at 4°C, Dec 0.5% chitosan was found to have the lowest TPC, followed by both K chitosan treatments. Insect chitosan coatings, regardless of the bleaching step, at the highest tested concentrations (1%), performed better than the negative control, proving to be the best treatments. Regarding the mixed storage condition, when treated with chitosan from *H. illucens*, the fruits showed the highest TPC, especially No Dec 0.5% and Dec 1%, proving the best coating solutions. The negative control and K, at both concentrations, had the lowest TPC (Table 3). Finally, a significant decrease in TPC was observed by crustacean-derived chitosan compared to chitosan from *H. illucens* both at controlled temperature and at mixed condition (RT + 4°C). Only at RT, crustacean chitosan showed values comparable to insect chitosan. A higher TPC is considered favorable, being an indicator of natural bioactive compounds that promote health.^68^ In general, a positive influence of chitosan coating was reported in several fruits, including strawberry.^19^^,^^51^^,^^69^ Similar results were reported by Petriccione et al. with significant TPC values compared to the untreated control, for the three strawberry cultivars, including “*Jonica*”, treated with 1 and 2% chitosan and stored at 4°C.^18^ The same was also described by Wang & Gao, who observed higher TPC and total flavonoid content (TFC) in strawberries coated with 0.5, 1 and 1.5% chitosan stored at 4°C and 10°C,^70^ with average TP values lower than ours, probably due to the different cultivars used in the experiments. Badawi et al., however, reported that chitosan, compared with the untreated control, was able only to contain the decrease in TPC measured in strawberries after 7 days at 4°C.^55^ In contrast to our results, at RT chitosan coating was effective in preserving the TPC of postharvest strawberries in comparison with untreated controls only when the coating solution was enhanced with turmeric and green tea extracts,^20^ or polyphenols derived from apple peel.^71^ Indeed, another novelty of our work is having tested the effect of chitosan coating not only at 4°C, i.e., the storage condition most commonly used in works on strawberries, but also at RT and at a mixed condition (4°C + RT), simulating retail sale at a supermarket.

#### Total flavonoid content and total anthocyanins

Flavonoids are an important group in the family of phenolic compounds with antioxidant and biological activity, contributing to the maintenance of physiological functions of plant species. Anthocyanins, water-soluble polyphenolic pigments of which strawberries are a rich source, belong to these compounds.^72^ Several authors have found that P3g, with contents varying up to 90%, is the most common anthocyanin in strawberry (*Fragaria x ananassa*) fruits, justifying its use as a reference standard.^73^ As for TP, current literature reports the effectiveness of chitosan coating in preserving TFC and TA during fruit storage.^35^^,^^56^ During the different storage conditions, as observed for TPC, also TFC and TA showed an increase after application of the different coating solutions. In strawberries stored at RT and 4°C, chitosan treatments showed no significant variations in TFC and TA compared to controls, although slight differences occurred. Notably, all coating treatments proved to preserve and contain flavonoid and anthocyanin concentrations during the ripening of the fruits (Table 3). In strawberries stored at mixed temperature condition, the TF and TA were significantly less concentrated in the negative control, solvent and K than in those treated with insect chitosan, which showed the highest values (Table 3). Indeed, under this condition, chitosan from *H. illucens*, particularly the No Dec at the highest concentration tested (1%), showed to preserve and increase both TFC and TA compared to the negative control during fruit ripening (Table 3). The same differences in the efficacy of insect chitosan compared to crustacean chitosan are evident. We can hypothesize that the lower Mw (about 70 kDa vs. 370 kDa, for chitosan from pupal exuviae of *H. illucens* and commercial one, respectively) may be responsible for its better activity.^35^ A Mw-related effect was reported by Jiang et al. who showed that chitosan coating with an Mw of 61 kDa maintained strawberry TFC reduction better (reduction from 18.24 to 30.92%) than uncoated fruits (reduction of 40%).^19^ Our results are partially consistent with Petriccione et al.*,*^18^ whose chitosan-coated fruits maintained higher flavonoid and anthocyanin contents than uncoated fruits only at cold storage. Chitosan treatment improved the nutraceutical properties of treated strawberries by maintaining high levels of phenols, flavonoids and also anthocyanin in postharvest; in addition, the reported results suggest an effect of chitosan during storage in slowing fruit senescence and improving phytochemical content.^70^ Unlike our results, as already reported for TPC, a positive influence of chitosan coating on strawberry TFC and TA were found only when the polymer was enriched with biologically active molecules.^20^^,^^71^

#### Antioxidant activity

Due to its antioxidant properties, chitosan, also in coating form, proved to be a valuable antioxidant agent promoting the shelf life of various fruits and vegetables, including the strawberries analyzed in this study.^51^^,^^69^^,^^70^ Antioxidant activity is an indicator of fruit health status correlated with the content of polyphenols and flavonoids. Indeed, our results showed a TAA trend similar to that of TPC, for all three storage conditions tested. Application of the different coatings induced a significant increase in the strawberry TAA, with values ranging from 7 to more than 12 mg TE g^−1^, depending on the concentration and storage conditions (Table 3). Particularly, strawberries stored at RT (9.3–11.44 mg TE g^−1^) and 4°C (8.3–10.8 mg TE g^−1^) showed less marked differences compared to the mixed condition 4°C + RT (7–12.4 mg TE g^−1^). After 4 days at RT, only No Dec 1%, as well as K 1%, showed the highest and significant TAA compared to the negative control, while the lowest TAA was observed for solvent and Dec 1% (Table 3). No Dec 1% showed the highest TAA in strawberries stored for two weeks at 4°C, followed by No Dec 0.5% and Dec 1% treatments, while the lowest activity was recorded for solvent and K treatments. As for TPC, in mixed storage condition, chitosan from *H. illucens* also showed the best coating solutions for antioxidant activity, especially No Dec 0.5% and Dec 1% chitosan. The negative control, solvent and K had the lowest TAA (Table 3). Both cold storage and mixed showed the significant decrease in TAA of crustacean chitosan compared to insect chitosan and negative control; it was not evident at RT, in which they had comparable activity on coated strawberries. Therefore, our results confirmed the better performance of chitosan from *H. illucens* also for this parameter, proving that it not only extended the shelf life, but also allowed good antioxidant activity to be preserved for prolonged times in coated strawberries, as already reported for tomatoes by Tafi et al*.*^35^ Similar results were reported by Petriccione et al.^18^ with significant TAA values compared to the untreated control for all tested strawberry cultivar, and particularly “*Jonica*”, treated with 1 and 2% chitosan after 6 days and more of storage at 4°C. At the same way, Wang & Gao observed an increase TAA in terms of DPPH scavenging capacity of strawberries coated with 0.5, 1 and 1.5% chitosan stored at controlled temperature, with the highest values recorded after 6 (at 10°C) and 9 days (at 4°C).^70^ In contrast to our results, as already reported for TPC and TFC, a positive effect of chitosan coating on strawberry TAA was noted exclusively when green tea extracts and polyphenols were added to chitosan coating solution.^20^^,^^71^

### Effect of chitosan coating on strawberry postharvest decay

The effect of coating on strawberry preservation, storing fruits for 4 days at RT, is shown in Table 4. No Dec chitosan was able to significantly reduce the disease incidence compared to both controls, negative and solvent-coated, at the highest concentration used. The other disease parameters, calculated by other indexes did not show significant differences, although treatments with No Dec chitosan, at both concentrations, induced the lowest McKinney^74^ index (MI) values (71.2 ± 7.2 and 72 ± 4.6, respectively). When the fruits were stored 14 days at 4°C, the effect of coating was more evident once again for No Dec 1% in the case of the disease incidence, but the significantly lowest MI was observed by treating the fruits with Dec 1% (Table 4). At the same time, chitosan from *H. illucens* showed a decreasing trend of DS and MI% values, although without significant differences, compared to the controls and K chitosan. The effect of mixed storage condition, cold storage for 7 days and then 3 days at RT, on strawberry preservation was shown in Table 4. Remarkably, Dec chitosan was able to significantly reduce the incidence (75 ± 10) and severity (2 ± 0.6) at the lowest and the highest concentration used, respectively. As a consequence, the MI had significantly lowest values in fruits coated with Dec chitosan samples, both 0.5 and 1%. Overall, coating with insect-derived chitosan on strawberries under different conditions of storage resulted in a reduction of fungal decay, probably gray mold and *Rhizopus* rot symptoms. Specifically, when strawberries were stored at RT for 4 days (Table 4), No Dec 1% induced an improvement in terms of incidence, which was reduced in fruit storage at 4°C for 14 days (Table 4). In this latter case, Dec 1% controlled the disease by reducing both the incidence and the severity. Although the decrease in values was not significant for disease incidence (DI) and disease severity (DS), Dec 1% allowed a significant reduction of the total MI compared to control. The situation changed by varying the storage conditions. Indeed, when fruits were stored at 4°C + RT (Table 4), Dec 1% showed the best ability to reduce decay on coated fruits. This coating chitosan sample always significantly reduced MI (31% and 29% for Dec 0.5% and Dec 1%, respectively), acting on different parameters depending on the used concentration: Dec 0.5% reduced the incidence by 20% compared to the negative control, while Dec 1% lowered the severity from class 3.6 (control) to class 2. Noteworthy, Dec chitosan showed MI values statistically lower than K chitosan, in agreement with data reported in a study where, under similar storage conditions, preharvest fruits were treated with commercial chitosan hydrochloride at a concentration of 0.5% (MI = 36.1%) and 1% (MI = 36.8%).^24^^,^^75^ This result supports the possibility of using chitosan derived from insects as a repressive compound to prevent and/or slowdown the appearance of phytopathogenic fungi during postharvest storage.

**Table 4:** Evaluation of postharvest decay of treated and untreated strawberries

Treatments	DI (%)	DS (1–5)	MI (%)
**RT**			

Ctrl -	100 ± 0^a^	3.7 ± 0.6^a^	74.7 ± 11.5^a^
Solvent	100 ± 0^a^	4.5 ± 0.6^a^	89 ± 11^a^
K 0.5%	100 ± 0^a^	4.1 ± 0.2^a^	81.3 ± 4.6^a^
K 1%	100 ± 0^a^	4.2 ± 0.7^a^	80 ± 10.6^a^
Dec 0.5%	100 ± 0^a^	3.8 ± 0.5^a^	76 ± 9.2^a^
Dec 1%	100 ± 0^a^	4 ± 0.3^a^	80 ± 6.5^a^
No Dec 0.5%	96 ± 8.9^ab^	3.7 ± 0.2^a^	71.2 ± 7.2^a^
No Dec 1%	85 ± 10^b^	4.3 ± 0.4^a^	72 ± 4.6^a^

**4°C**			

Ctrl -	66.7 ± 16.7^ab^	2.1 ± 0.5^a^	28.9 ± 13.5^ab^
Solvent	83.3 ± 13.6^a^	2.2 ± 0.6^a^	36.7 ± 9.8^a^
K 0.5%	70.8 ± 16^ab^	1.9 ± 0.2^a^	27.5 ± 7.9^ab^
K 1%	77.8 ± 9.6^ab^	2.1 ± 0.3^a^	32.2 ± 7.7^ab^
Dec 0.5%	72.2 ± 9.6^ab^	1.5 ± 0.3^a^	21.1 ± 3.8^ab^
Dec 1%	58.3 ± 9.6^ab^	1.5 ± 0.1^a^	17.5 ± 3.2^b^
No Dec 0.5%	77.8 ± 9.6^ab^	1.5 ± 0.3^a^	23.3 ± 3.3^ab^
No Dec 1%	54.2 ± 8.3^b^	1.8 ± 0.6^a^	19.2 ± 5.7^ab^

**4°C + RT**			

Ctrl -	95 ± 10^ab^	3.6 ± 0.3^a^	67 ± 2^a^
Solvent	100 ± 0^a^	2.8 ± 1^ab^	55 ± 20^ab^
K 0.5%	100 ± 0^a^	2.9 ± 0.3^ab^	57 ± 6.8^ab^
K 1%	95 ± 10^ab^	3.8 ± 0.4^a^	72 ± 11.8^a^
Dec 0.5%	75 ± 10^b^	2.4 ± 0.6^ab^	36 ± 11.8^b^
Dec 1%	92 ± 11^ab^	2.0 ± 0.6^b^	37.6 ± 12.5^b^
No Dec 0.5%	92 ± 11^ab^	2.9 ± 0.8^ab^	52.8 ± 12.5^ab^
No Dec 1%	90 ± 11.5^ab^	2.8 ± 0.5^ab^	49.6 ± 14.6^ab^

Disease incidence (DI), disease severity (DS), and McKinney index (MI%) of postharvest decay of strawberry fruit stored at RT, 4°C and mixed condition (4°C + RT). Treatments: negative control (Ctrl -), solvent, coating with pupal exuviae not decolorized (No Dec), decolorized (Dec) and commercial (K) chitosan, both at 0.5% and 1%. Data are expressed as mean ± standard deviation (n = 5). Means followed by different letters in the column are significantly different (p < 0.05) by one-way ANOVA and Tuckey *post-hoc* test.

### Conclusions

Strawberries are one of the most popular fruits among consumers, however, they are also one of the most perishable. When stored under normal conditions, the fruit retains its integrity after harvest for only a few days. To slow down this decay process and increase the fruit shelf life, a treatment with biodegradable, health-friendly compound, such as chitosan, was proposed as option. This bio-coating would act as a protective barrier against both external agents and molds or fungi that could lead to fast fruit decay. In literature, there are already studies investigating the action of chitosan on strawberry preservation. However, they use commercial chitosan produced from crustaceans and, to date, there is no mention concerning insect chitosan. To the best of our knowledge, the present work is the first one in which chitosan from *H. illucens* is applied as a coating on strawberries. In addition, chitosan from *H*. *illucens* is applied to a new strawberry cultivar, the Lucanian *“Melissa”*, and this is also the first study in which the decay of this new fruit is observed. Our findings showed that *H. illucens* chitosan was effective in food preservation, especially in stabilizing some crucial postharvest parameters. In particular, Dec chitosan was most effective in containing physico-chemical parameters (0.5% for WL and 1% for SSC) and in preserving treated strawberries from fungal decay; No Dec chitosan, on the other hand, was more functional in preserving and enhancing the nutraceutical value of the treated strawberries. Further studies need to understand if, modifying the chitosan properties or varying chitosan concentrations, the coating effect could be further enhanced. These findings, although preliminary, are a solid starting point for validating insect biomasses as source of chitosan to be used in the agri-food chain.

## STAR★Methods

### Key resources table

**Table:** 

REAGENT or RESOURCE	SOURCE	IDENTIFIER
**Chemicals, peptides, and recombinant proteins**		

Chitosan	Sigma-Aldrich Co.	CAS: 9012-76-4
Hydrochloric acid	Sigma-Aldrich Co.	CAS: 7647-01-0
sodium hydroxide	Sigma-Aldrich Co.	CAS: 1310-73-2
Acetic acid	Sigma-Aldrich Co.	CAS: 64-19-7
2,2-diphenyl-1-picrylhydrazyl (DPPH)	Sigma-Aldrich Co.	CAS: 1898-66-4
potassium ferricyanide	Sigma-Aldrich Co	CAS: 13746-66-2
sodium phosphate buffer	Sigma-Aldrich Co	MFCD00131855
trichloroacetic acid	Sigma-Aldrich Co	CAS: 76-03-9
ferric chloride	Sigma-Aldrich Co.	CAS: 7705-08-0
glycerol	Sigma-Aldrich Co.	CAS: 56-81-5
Tween-80	Sigma-Aldrich Co	CAS: 9005-65-6
Methanol	Sigma-Aldrich Co.	CAS: 67-56-1
Folin–Ciocalteau	Sigma-Aldrich Co.	1.09001
Gallic acid	Sigma-Aldrich Co	CAS: 149-91-7
sodium nitrate	Sigma-Aldrich Co	CAS: 7631-99-4
Aluminum chloride	Sigma-Aldrich Co	CAS: 7446-70-0
Quercetin	Sigma-Aldrich Co	CAS: 849061-97-8
potassium chloride	Sigma-Aldrich Co.	CAS:7447-40-7
sodium acetate	Sigma-Aldrich Co.	CAS: 127-09-3
ABTS	Sigma-Aldrich Co	CAS: 30931-67-0
Trolox	Sigma-Aldrich Co	CAS: 53188-07-1

**Experimental models: Organisms/strains**		

Pupal exuviae of *Hermetia illucens*	*Hermetia illucens* farming, Xflies s.r.l (Potenza, Italy) and Protix (Dongen, The Netherlands)	https://protix.eu/
*Fragaria x ananassa cv. Melissa*	APOFRUIT Italia soc. coop. agricola, Scanzano Jonico (Matera, Italy)	Copyright © 2022 Nova Siri Genetics https://www.novasirigenetics.com/

**Software and algorithms**		

Prism (GraphPad Software)	GraphPad	https://www.graphpad.com/

### Resource availability

#### Lead contact

All requests for additional information and resources should be directed to patrizia.falabella@unibas.it.

#### Materials availability

All material used in this study are available from the lead contact upon request.

#### Data and code availability

All data reported in this paper will be shared by the lead contact upon request. This paper does not report original code. Any additional information required to reanalyze the data reported in this paper is available from the lead contact upon request.

### Experimental model and study participant details

#### Pupal exuviae of *Hermetia illucens*

Chitosan was produced from pupal exuviae of *H. illucens* kindly provided by Xflies s.r.l (Potenza, Italy) and Protix (Dongen, The Netherlands).

#### Fragaria x ananassa cultivar “Melissa”

New local strawberry (*Fragaria x ananassa cv. Melissa*) (Copyright © 2022 Nova Siri Genetics), belonging to the Rosaceae family, were supplied by a local grower (APOFRUIT Italia soc. coop. agricola, Scanzano Jonico, Matera, Italy). The fruit were graded according to similar size, shape, color and ripeness degree, as well as the absence of visible fungal infections and mechanical defects.

### Method details

#### Production and characterization of chitosan from *H. illucens*

Pupal exuviae from *H. illucens* were used to extract both not decolorized and decolorized chitin. Both chitins were subjected to a heterogeneous deacetylation process, as reported in Triunfo et al*.*^27^ Not decolorized (No Dec) and decolorized (Dec) chitosan from pupal exuviae was chemically examined by Fourier-transform infrared spectroscopy (FTIR) and X-ray diffraction (XRD) analysis to confirm its identity and purity, as reported in Triunfo et al.^27^^,^^36^ For each chitosan sample, the deacetylation degree (DD), the viscosity-average molecular weight (M_v_) by intrinsic viscosity and the film-forming ability were determined.^35^^,^^36^

#### Determination of chitosan antioxidant properties

The antioxidant activity of chitosan from *H. illucens* was evaluated according to different tests, described below.

##### Scavenging ability on 2,2-diphenyl-1-picrylhydrazyl (DPPH) radicals

The free radical scavenging activity of chitosan samples from *H. illucens* was determined. For each chitosan different concentrations (5, 2.5, 1.25, 0.625, 0.3125, 0.156 mg/mL) were tested. 2,2-diphenyl-1-picrylhydrazyl was solubilized in methanol to obtain a solution of 6·10^-5^ M and added to No Dec and Dec chitosan samples. Solutions were incubated for 30 min in the dark at room temperature and the absorbance was measured at 517 nm with a spectrophotometer (Thermo Scientific Multiskan Go). The antioxidant activity was expressed as IC_50_ value (mg/mL), the concentration of chitosan at which DPPH radicals were scavenged by 50%.

##### Ferric reducing antioxidant power (FRAP)

The reducing power of chitosan samples from *H. illucens* was estimated. The same concentrations as above of chitosan were mixed with 0.2 M sodium phosphate buffer and 1% potassium ferricyanide solution and were incubated at 50°C for 20 min. After the incubation, 10% trichloroacetic acid was added and the mixtures were centrifuged for 5 min. Absorbance was measured at 700 nm with a spectrophotometer (Thermo Scientific Multiskan Go) after addition of distilled water and 0.1% ferric chloride. The antioxidant activity was expressed as EC_50_ value (mg/mL), the concentration providing the absorbance at 0.5 nm. As above, ascorbic acid was used as a positive control.

#### Formulation and application of insect-derived chitosan coating solutions

Briefly, chitosan from *H. illucens* and commercial chitosan were dissolved in a solvent solution consisting of 1% acetic acid, with addition of 2% glycerol and 0.2% Tween-80, in order to prepare two solutions of 0.5% and 1% (w/v) for each chitosan sample tested (commercial chitosan, No Dec and Dec chitosan from pupal exuviae). A treatment with solvent solution without chitosan and a negative control (strawberries without treatment) were also carried out. There are 8 experimental conditions: (1) negative control (untreated fruit), (2) solvent treatment, (3) 0.5% and (4) 1% commercial chitosan, (5) 0.5% and (6) 1% Dec chitosan, (7) 0.5% and (8) 1% No Dec chitosan.

For each coating solution, the kinematic viscosity was measured, and the pH was adjusted to prevent its precipitation at alkaline pH, as reported in Tafi et al*.*^35^ Chitosan coating solutions were applied by spraying through an aerograph (Martellato s.r.l., Rovigo, Italy) taking care to provide uniform surface coverage of the treated fruits. For a more comprehensive evaluation, the strawberry storage, which was carried until decomposition, was tested simultaneously under different conditions, specifically a part of the fruit was stored at controlled temperature (4°C) for 14 days, another part was kept at room temperature (RT) for 4 days. A third group of fruits was stored first at 4°C for 7 days and then at RT, all until decay, to simulate the storage conditions of the refrigeration chain, followed by retail sale.

#### Assessment of physicochemical properties of strawberry fruit

##### Weight loss

All fruits were weighed before and after storage using an electronic weighing balance (Sartorius- BCE ENTRIS II, Göttingen, Germany). The weight loss was expressed as percentage loss of the original fresh weight. In the case of the mixed condition, 4°C + RT, partial weight losses were also calculated.

##### Soluble solids content

The total soluble solids content (SSC) of the fruit pulp was determined using a digital refractometer (Lab Logistics Group GmbH, Meckenheim, Germany), according to the standard method EN ISO 2173:2003 and expressed as Brix°. Measured at the start and end of the experiment, the fluctuation of the SSC parameter during the storage period was evaluated.

##### pH

The pH of the fruit pulp was measured at RT with a pH meter (Orion Research Inc., Boston, USA). As with SSC, the variation during the storage period was also considered for this parameter.

#### Extraction and quantification of total phenolic and total flavonoid content, total anthocyanins and total antioxidant activity

Fruit samples were extracted with 80% methanol and the mixture was sonicated and then stirred for 1 h total. The mixture was filtered and then centrifuged (10 min, 5000 g) at 4°C and the supernatant was recovered and stored at 4°C. The pellet was re-extracted twice using 80% methanol. The resulting supernatants were used for the determination of the total phenolic, flavonoid and anthocyanins concentration and total antioxidant activity.

##### Total phenolic content

The total phenolic content (TPC) in the strawberry fruits was determined by the Folin–Ciocalteau reagent,^35^ recording the absorbance at 723 nm after 1 h of incubation in the dark. The results were expressed as mg of gallic acid equivalent (GAE) g^-1^ of fresh weight (FW) using a calibration curve of gallic acid standard (0–250 mg L^-1^).

##### Total flavonoid content

Total flavonoid content (TFC) was determined by the AlCl_3_ method.^63^ Each extract was added to 5% sodium nitrate. After 5 and 6 min, 10% AlCl_3_ and 1M sodium hydroxide solutions were added, respectively. Finally, distilled water was added up to 1.5 mL, and the absorbance was measured at 510 nm after 10 min and the results were expressed as mg quercetin equivalent (QE) g^-1^ FW using a calibration curve of quercetin standard (0–125 mg L^-1^).

##### Determination of total anthocyanins

Total anthocyanins (TA) were determined by the pH differential method.^64^ Absorbance of the mixture was measured at λ_vis-max_ and at 700 nm in 2 different buffers, 0.025M potassium chloride (pH 1.0) and 0.4M sodium acetate (pH 4.5). The results were expressed as mg of pelargonidin-3-glucoside (P3g) g^-1^ FW.

##### Total antioxidant activity

The ABTS assay was used to evaluate the total antioxidant activity (TAA) of the strawberry extract.^66^ The ABTS^+•^ solution was produced, diluted to an absorbance of 0.7 and mixed with each sample in order to produce between 20–80% inhibition of the blank absorbance at 734 nm after 30 min of incubation. The results were expressed as mg of Trolox equivalent (TE) g^-1^ FW, using a calibration curve of Trolox standard (0-125 mg L^-1^).

#### Evaluation of fungal decay

A decay study was conducted to verify the effect of No Dec and Dec chitosan from pupal exuviae and commercial chitosan in all tested storage conditions. At the observation time, disease severity was recorded according to an empirical scale with six degrees: 0, healthy fruit; 1, 1–20% fruit surface infected; 2, 21–40% fruit surface infected; 3, 41–60% fruit surface infected; 4, 61–80% fruit surface infected; 5, more than 81% of the strawberry surface infected. The disease incidence (DI), disease severity (DS) and McKinney index (MI), the latter including information on both DI and DS, were calculated according to McKinney^74^ and Romanazzi et al*.*^75^

### Quantification and statistical analysis

Each trial contained five replicates of six strawberries. All measurements were performed in triplicate and data were expressed as average ± standard deviation. Data were analyzed by one-way Anova and Tukey’s *post-hoc* test. Statistical analyses were performed using a GraphPad Prism version 6.0.0 for Windows (GraphPad Software, San Diego, California USA).
